# Supplementary material for: Persulfidation of DJ-1: Mechanism and Consequences
Source: Biomolecules. 2022 Dec 22;13(1):27. doi: 10.3390/biom13010027 (PMC9856005; doi:10.3390/biom13010027)

Figure S1. Kinetics of DJ-1 inactivation by sodium disulfide - Absorbance at 405 nm corresponding to the phenoxide released upon hydrolysis of pNPA (see the experimental section for conditions) recorded over time at 25°C after addition of DJ-1 (625 nM) to a solution of pNPa (2.8 mM) containing various concentrations of Na_2S_2.

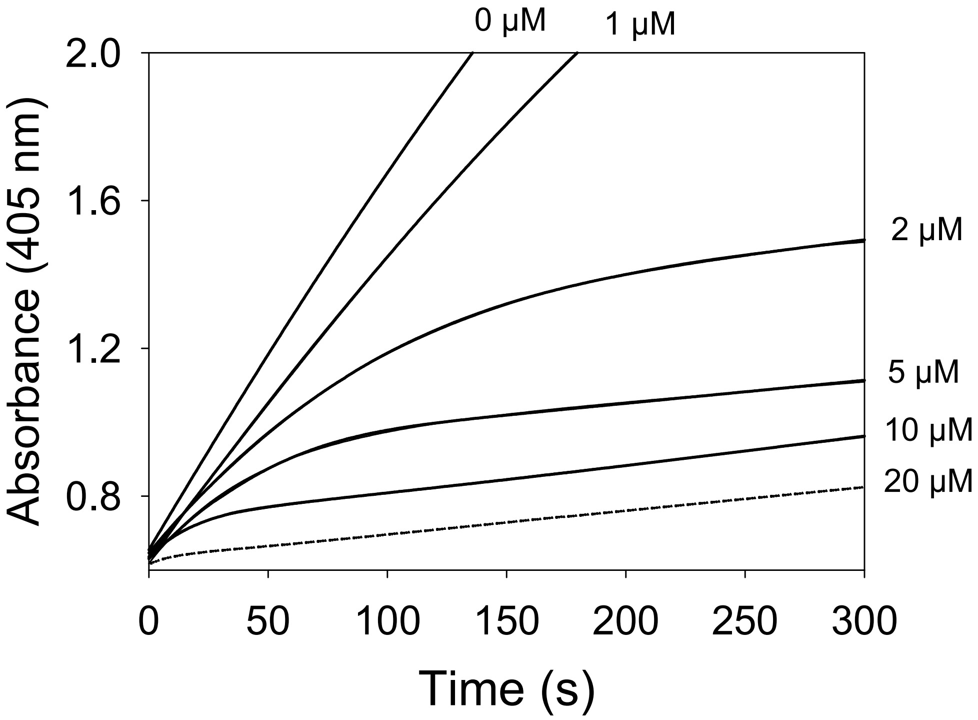

Figure S2. Detection of persulfidated DJ-1 - In-gel fluorescence (Cy3-canal (A) or Cy5-canal (B)) and Coomassie Brilliant Blue staining scans obtained from (A) purified WT or mutant C106S using the protocol from [85] or (B) from *E. coli* lysate using the protocol from [30].

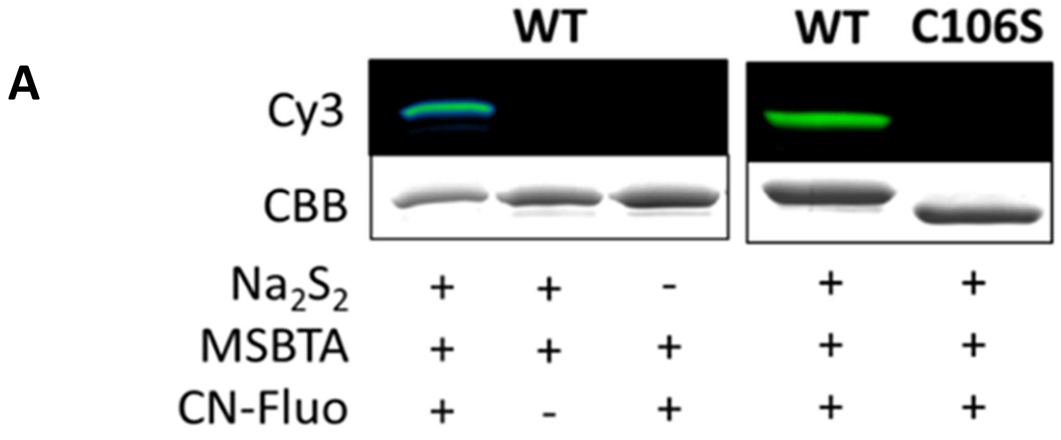

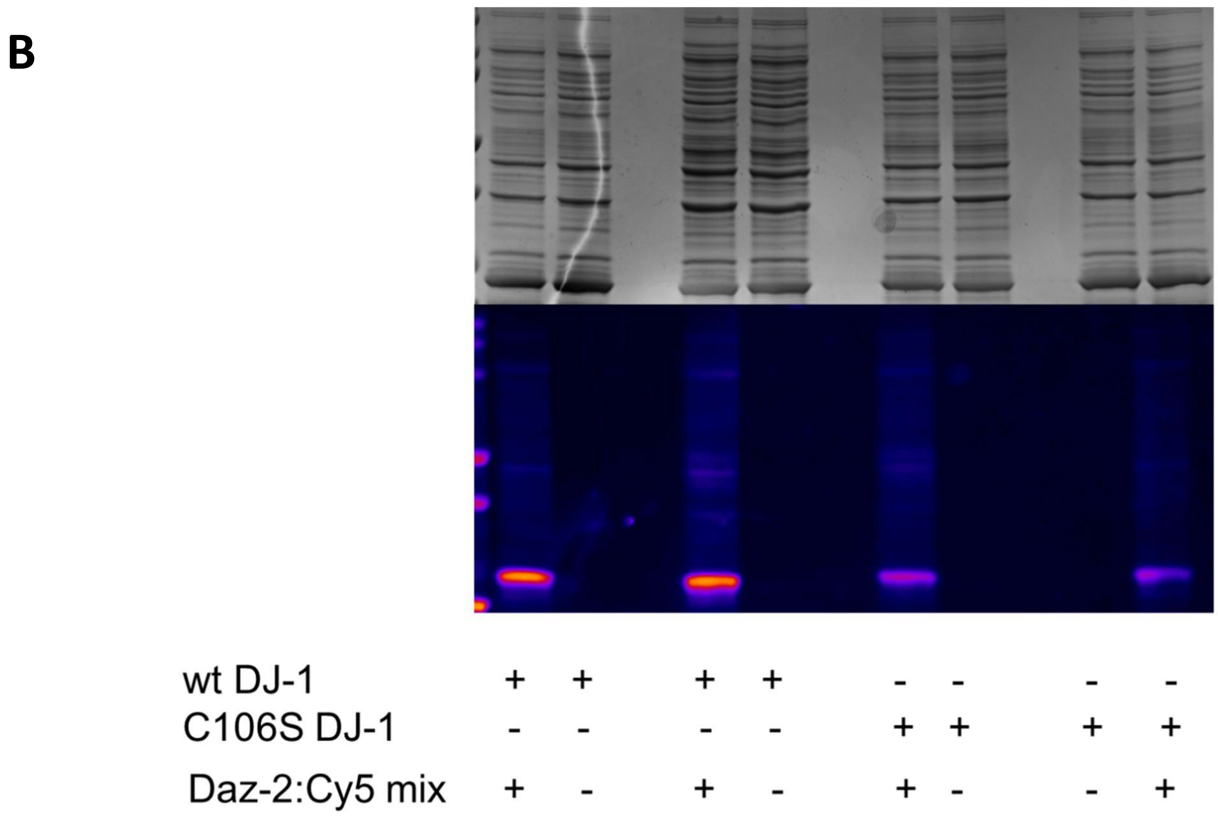

Figure S3. Final conformations of wt and persulfidated DJ-1 – Structural comparison of representative snapshots of wt (pink and light blue) and persulfidated (red and dark blue) DJ-1. The two main regions showing important differences in RMSD are highlighted in yellow (wt) and orange (persulfidated) for residues #106-#109, and green (light for the wt, dark for the persulfidated form) for residues #128-#138.

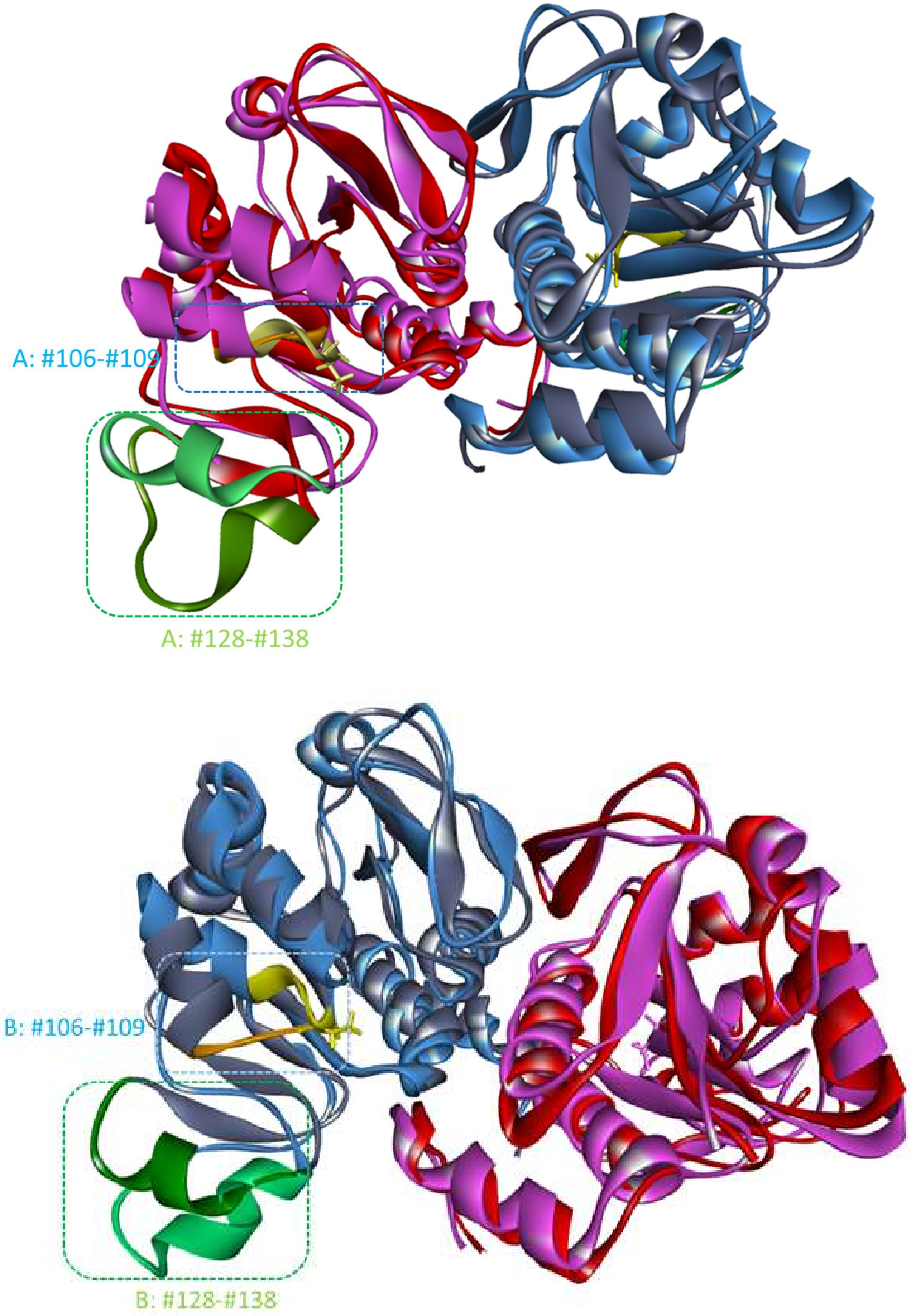

Figure S4. The main interaction between C106 and its neighbors are conserved upon persulfidation – Average distances (Å) recorded over the last 45 ns of the dynamics between C106 and interacting residues. Views of the amino-acids interacting with C106.

	d1(Å)		d2(Å)		d3(Å)		d4(Å)	d5(Å)		d6(Å)	
	OH(E18)…S(C106)		NH(G75)…O=C(C106)		NH(G75)…S(C106)			NH(S155)…O=C(C106)		NH(R156)…O=C(C106)	
	E18(A)	E18(B)	G75(A)	G75(B)	G75(A)	G75(B)	G108(B)	S1555(A)	S155(B)	R156(A)	R156(B)
wt	5.96	2.44	5.62	5.42	5.84	2.35	/	2.01	2.26	3.23	2.45
persulfidated	3.71	4.08	3.30	5.08	4.35	4.28	3.25	2.62	2.12	4.04	3.12

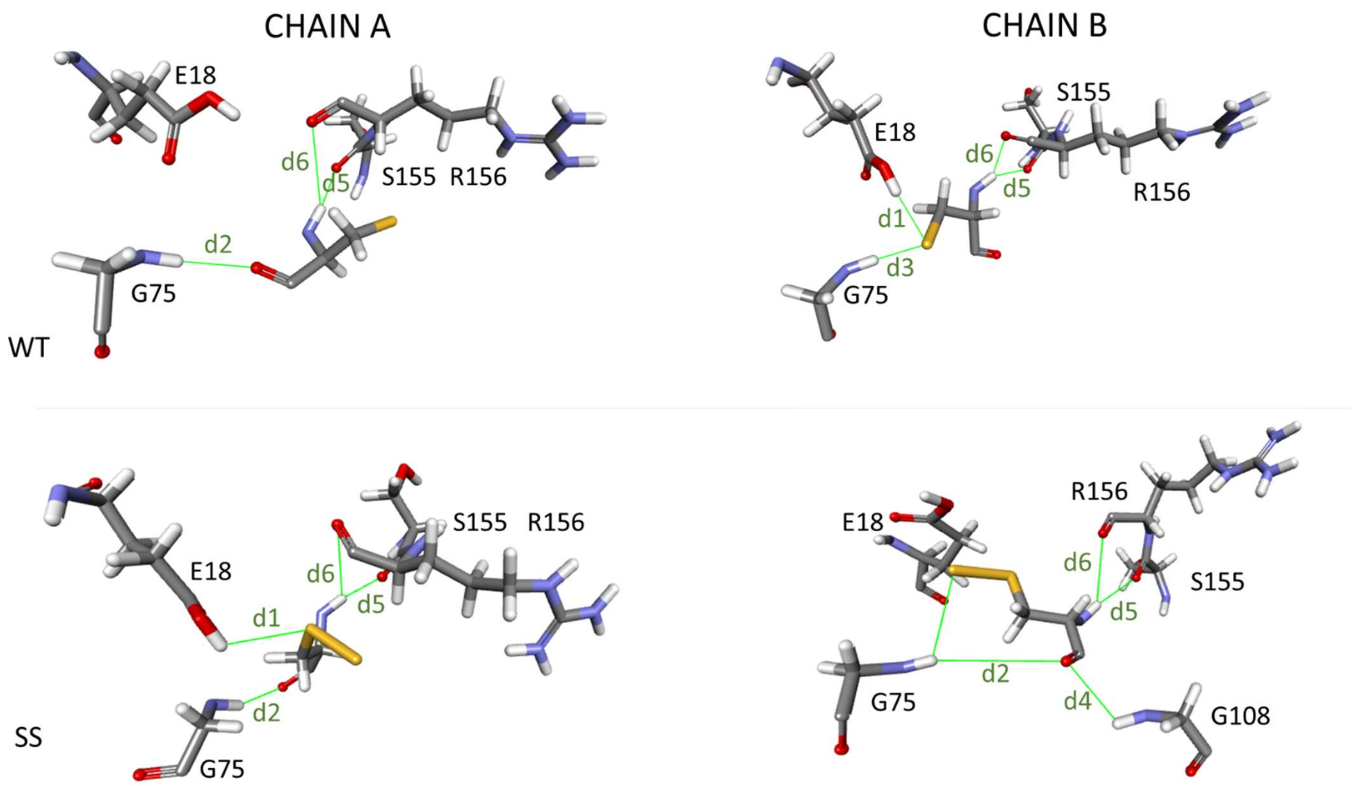

Figure S5. The interfacial H-bond intercation between R27:A and R48:B is lost after persulfidation – A) Representative snapshots obtained after 70 ns MD simulations of the wt (up) and persulfidated DJ-1 highlighting the loss of the interfacial H-bond interaction between R27 side chain (subunit A) and R48 backbone(subunit B), B) Time evolution of the distance between the centroid of the guanidine group of R27 and the carbonyle group of R48 over the course of the simulation in the wt (blue) and persulfidated (red) protein (bottom).

A

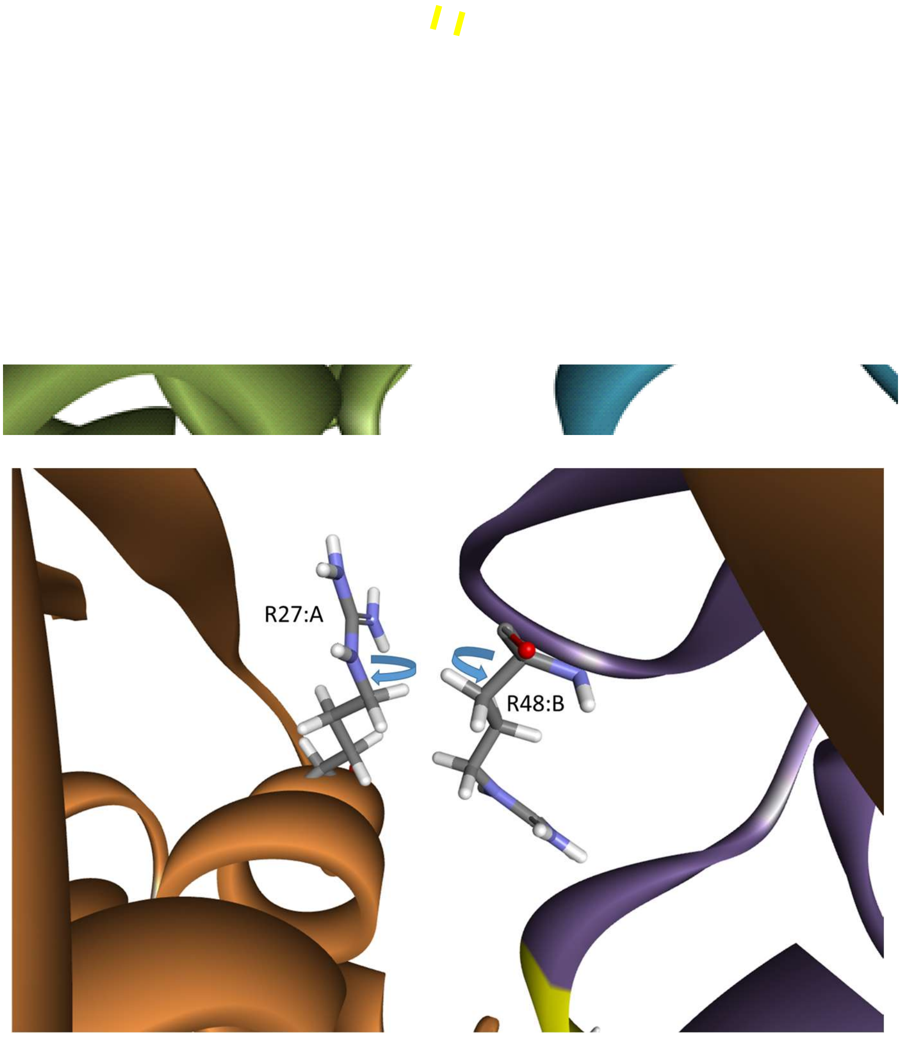

B

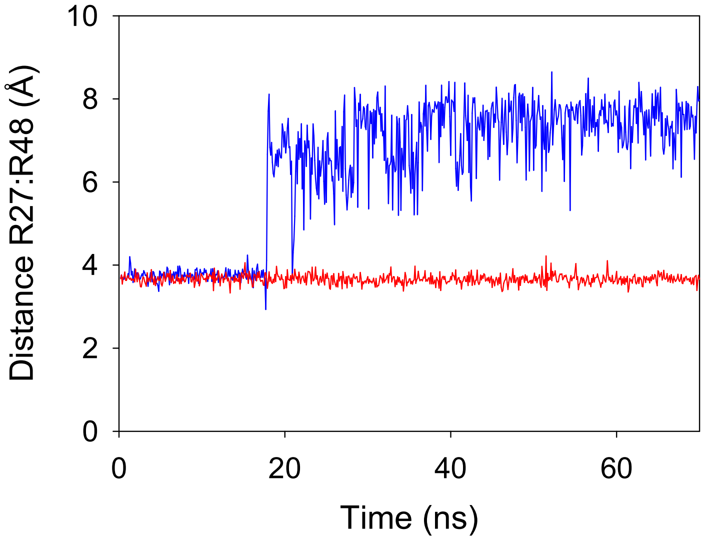

Figure S6. The interfacial H-bond interaction between G159:A and L185:B is lost after persulfidation – A) Representative snapshots obtained after70 ns MD simulations of the wt (up) and persulfidated DJ-1 highlighting the loss of the interfacial H-bond interaction between G159 and L185, B) Time evolution of the distance between the N-H group of G159 (subunit A) and the carbonyle group of L185 (subunit B) over the course of the simulation in the wt (blue) and persulfidated (red) protein.

.

A

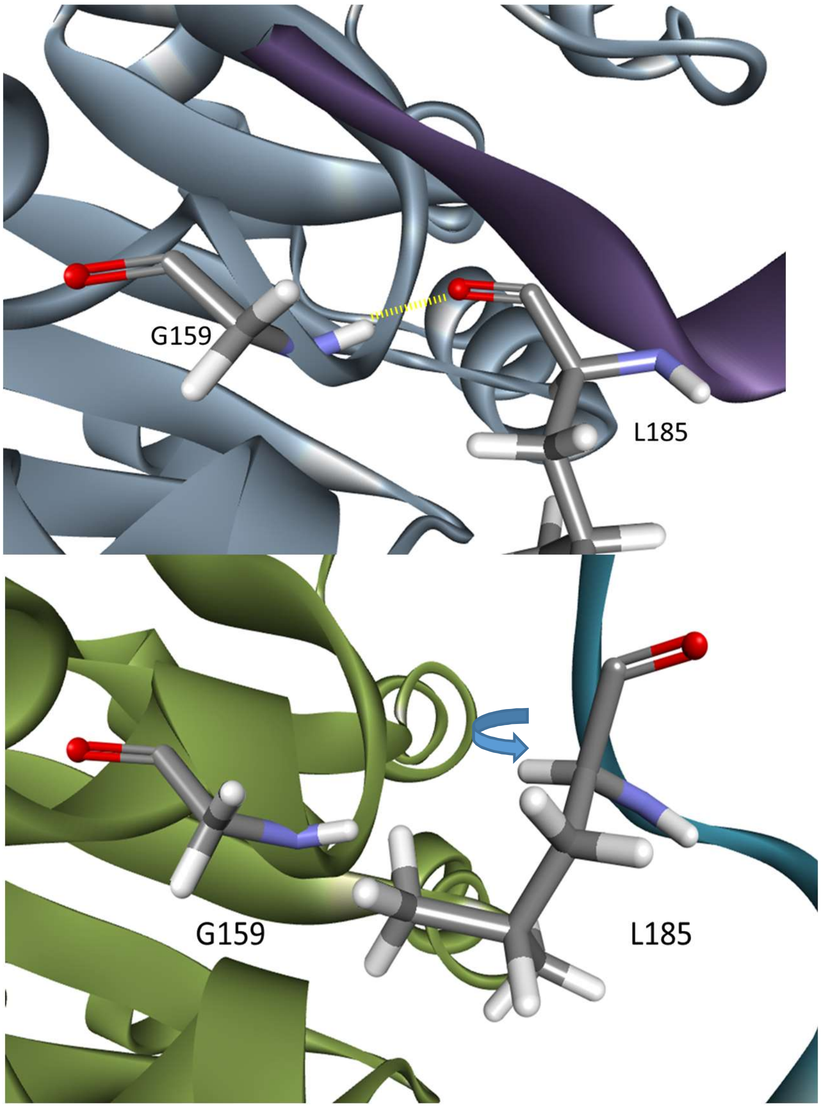

B

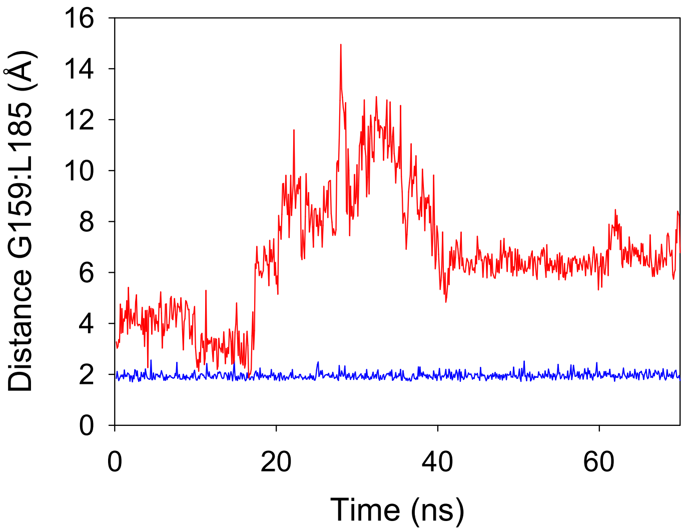

Figure S7. The interfacial interaction between R28:A and E15:B is weakened after persulfidation – A) Representative snapshot obtained after 70 ns MD simulations of the interfacial interaction between R28 and E15 (circled in yellow). B) Time evolution of the distance between the centroid of the guanidine group of R28 and the centroid of the carboxylate of E15 over the course of the simulation in the wt (blue) and persulfidated (red) protein.

A

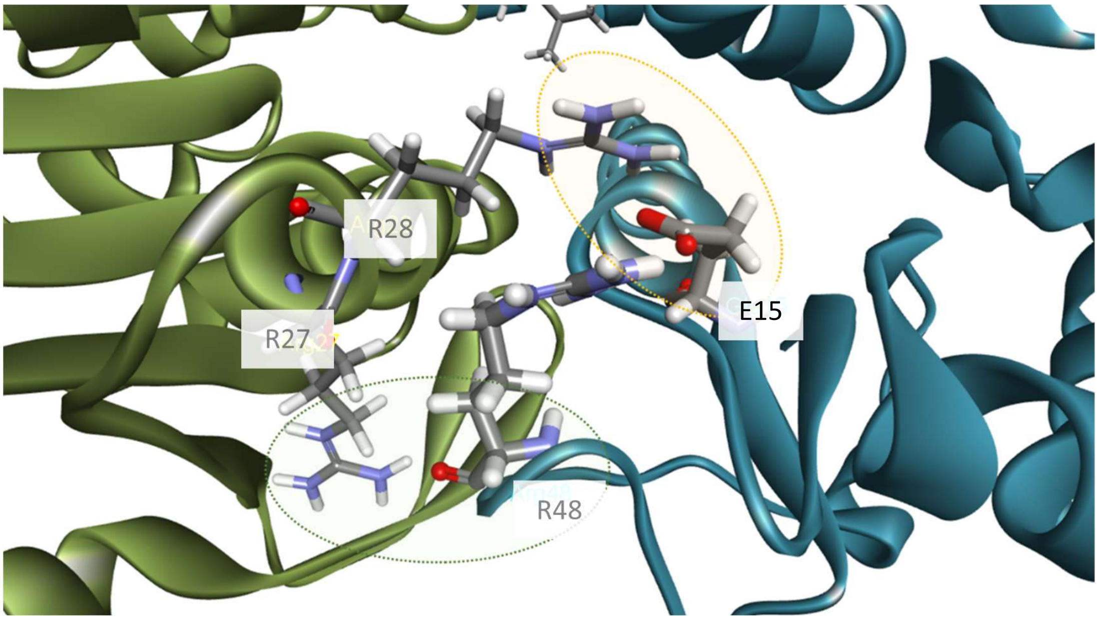

B

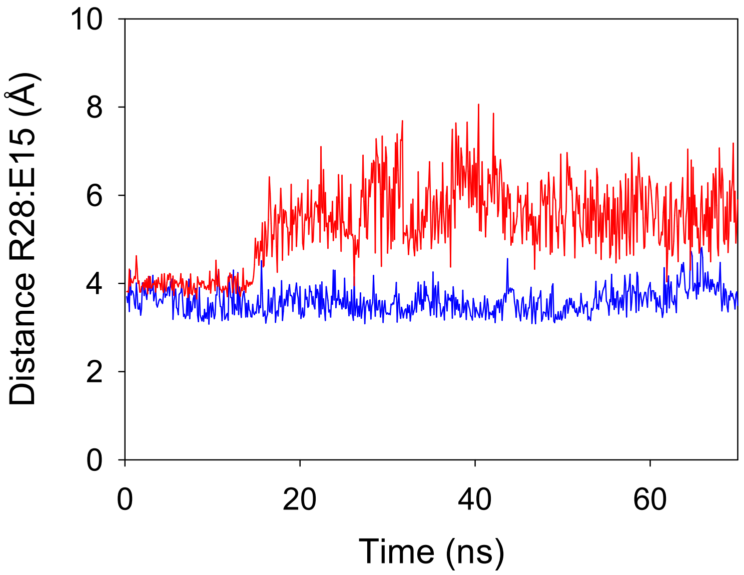

Figure S8. The interfacial interactions between E18:A and R28:B and between D49:A and R27:B are weakened after persulfidation – A) Representation of the interfacial interaction between E18 and R28 and between D49 and R27, B) Time evolution of the distance between the OH group of D18 and the centroid of the guanidine group of R28 (left) or between the centroid of the carboxylate groups of D49 and the centroid of the guanidine group of R27 (right) over the course of the simulation in the wt (blue) and persulfidated (red) protein.

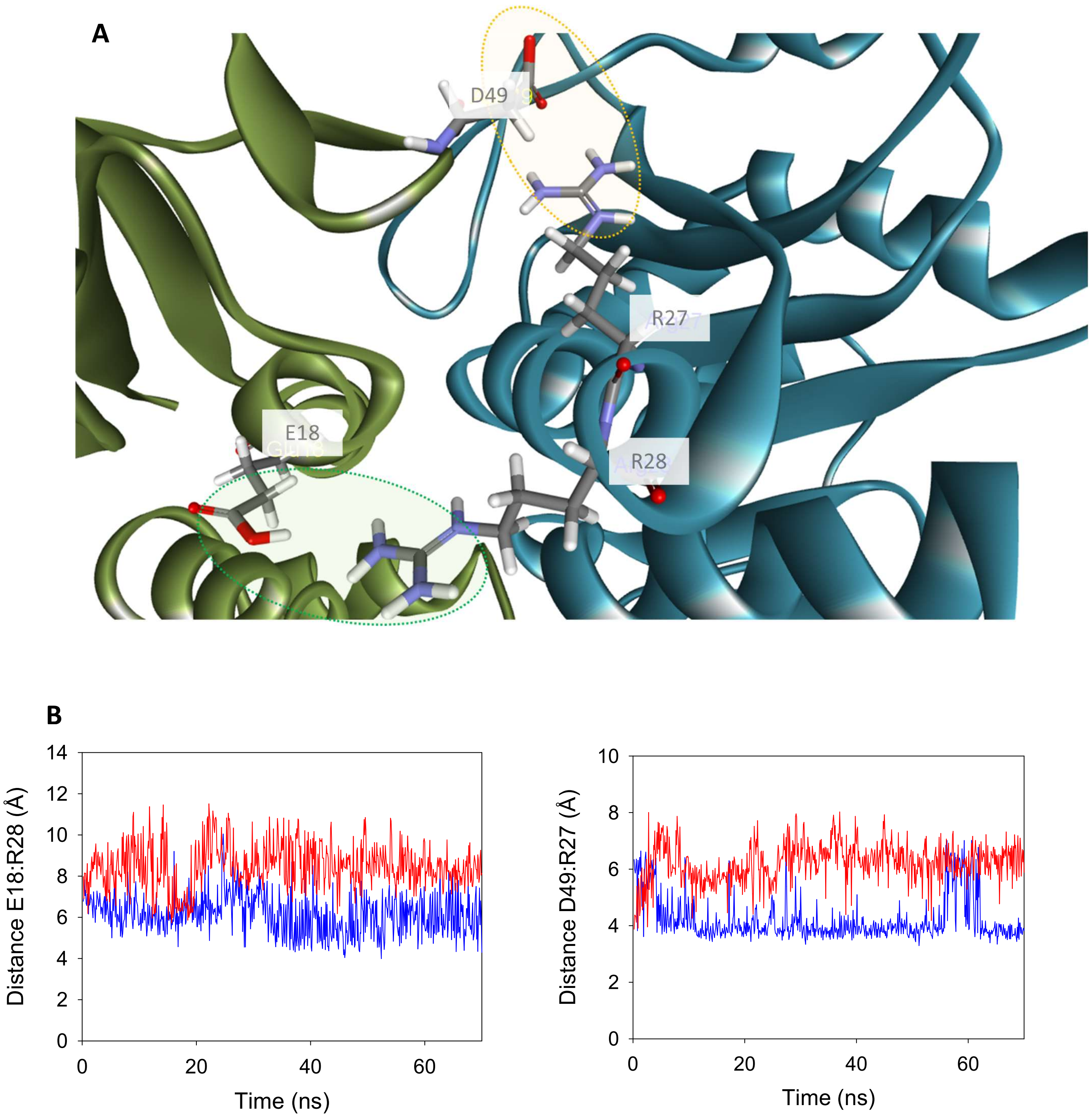

Figure S9. The interfacial interaction between S47:A and R28:B is strengthened after persulfidation – A) Representative snapshot obtained after 70 ns MD simulations of the interfacial interaction between S47 and R28, B) Time evolution of the distance between H-O group of S47 and the centroid of the $NH_{21/22}$ from the guanidine group of R28 over the course of the simulation in the wt (blue) and persulfidated (red) protein.

A

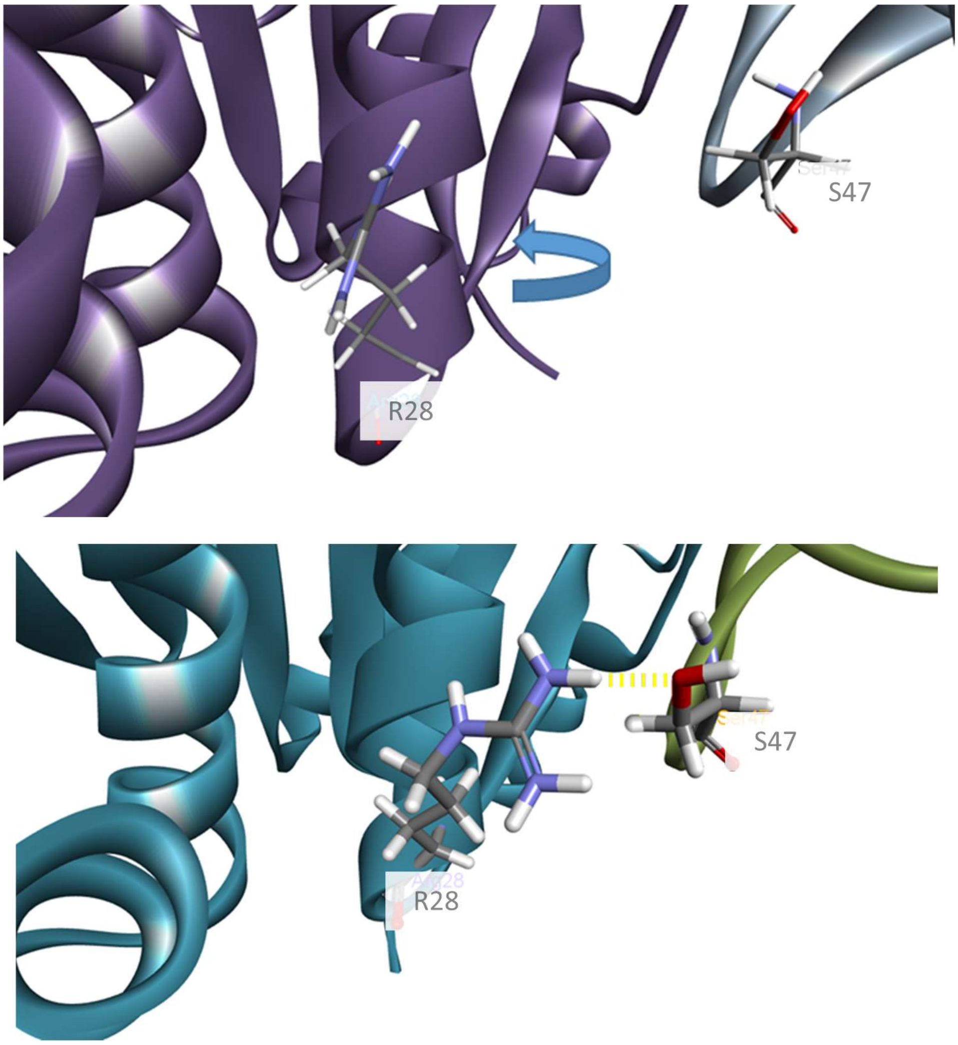

B

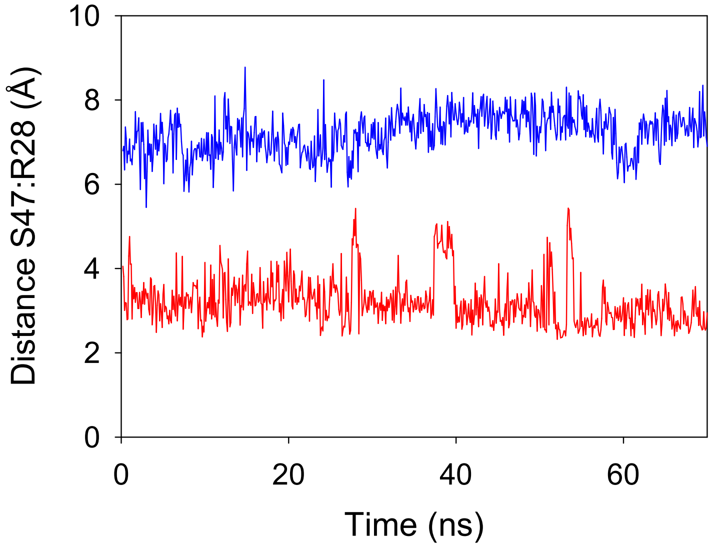

Figure S10. Persulfidation of DJ-1 induces changes at the protein surface – Electrostatic surface representation (left) and hydrophobicity surface (right) for both subunits of wt and SS-DJ1 after 70ns MD simulations. Electrostatic potential is colored to show negative charge in red and positive charge in blue. Hydrophobic and hydrophilic regions are representated in brown and blue, respectively. The localization of the protein subunits and of the sulfur atoms (yellow balls) are shown in the middle.

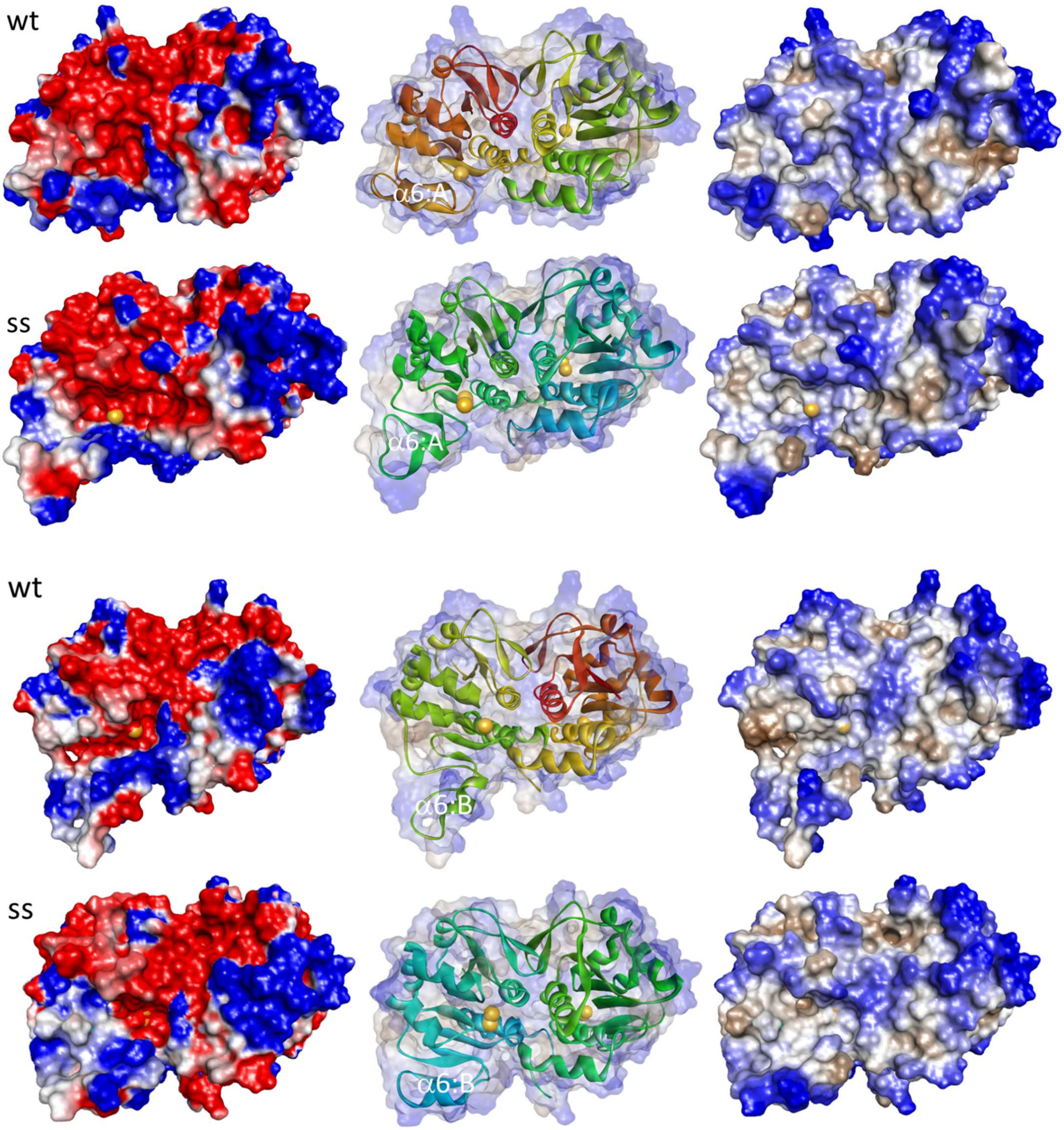

Figure S11. Structural comparison of sulfinylated and persulfidated DJ-1 – A) Structural comparison of representative snapshots obtained after 60 ns MD simulations the persulfidated (yellow) and sulfinylated (purple and brown) DJ-1. The main region showing important differences in RMSD is highlighted. B) Root-mean-square deviation (RMSD) plot measured on the protein backbone backbone for the sulfinylated DJ-1 during the MD simulations with 100 ps interval for the sulfinylated DJ-1, and Cα-Root Mean Square Fluctuations (RMSD) plots for the persulfidated (red) and sulfinylated DJ-1 (green, average value of the individual values of the monomers). RMSD plots represent the average of the RMSD value of each monomer.

A

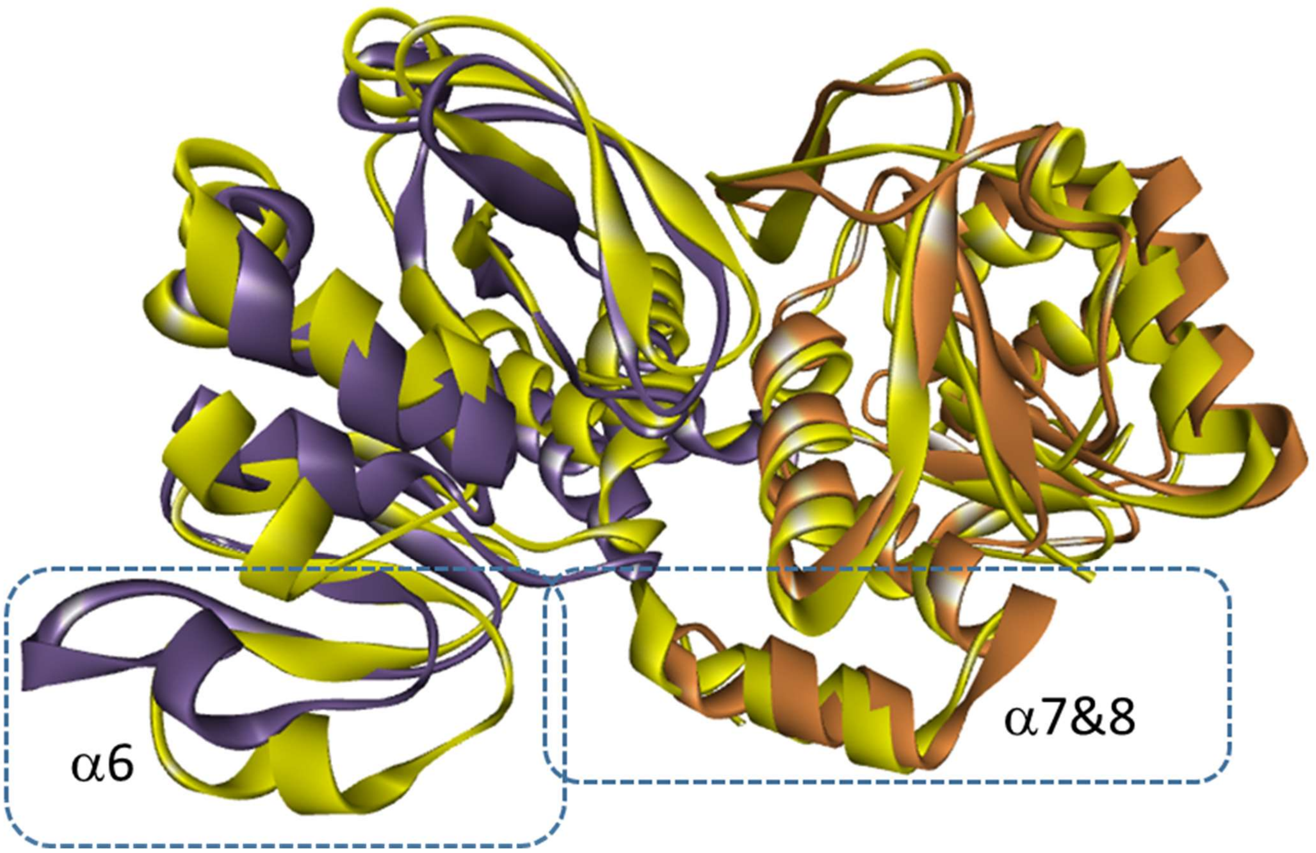

B

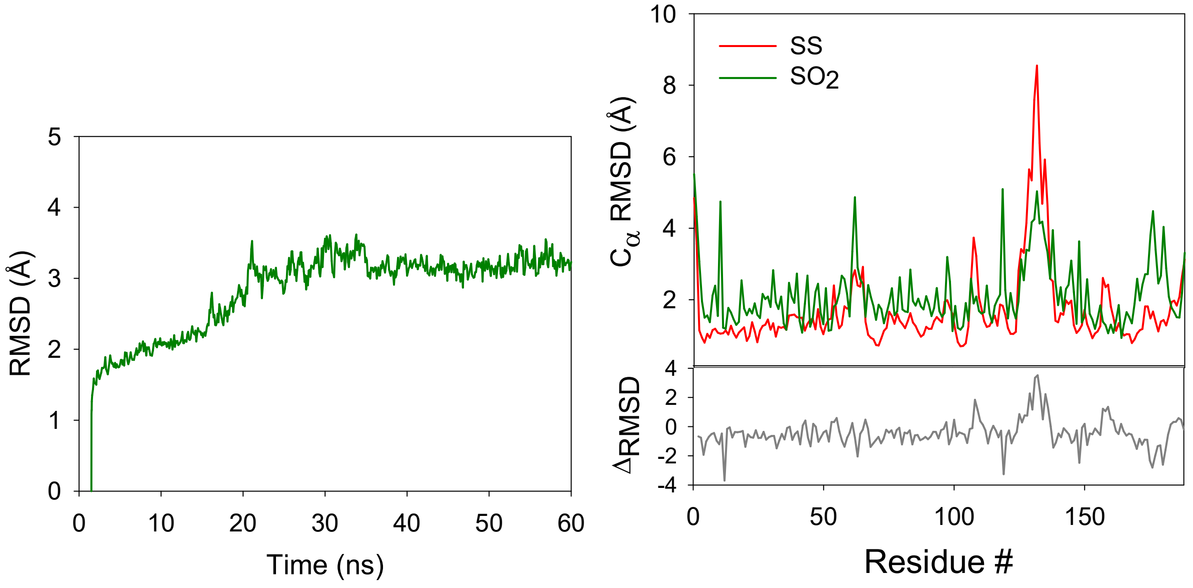

Figure S12. Main interactions between C106 and its neighbors in sulfinylated DJ-1 – Average distances (Å) recorded over the last 45 ns of the dynamics between C106 and interacting residues. Distances are O-H(E18)…O=S(C106), N-H(G75)…O=C(C106), N-H(G75)…S(C106) (*), N-H(S155 or R156)…O=C(C106), N-H(H126)…O=C or O=S(C106). Views of the amino-acids interacting with C106.

	E18(A)	E18(B)	G75(A)	G75(B)	H126(A)	H126(B)	S1555(A)	S155(B)
sulfenylated	2.42	5.49	5.13	5.85	2.05	3.22	4.48	2.01
persulfidated	3.71	4.08	3.30	5.08			2.62	2.12

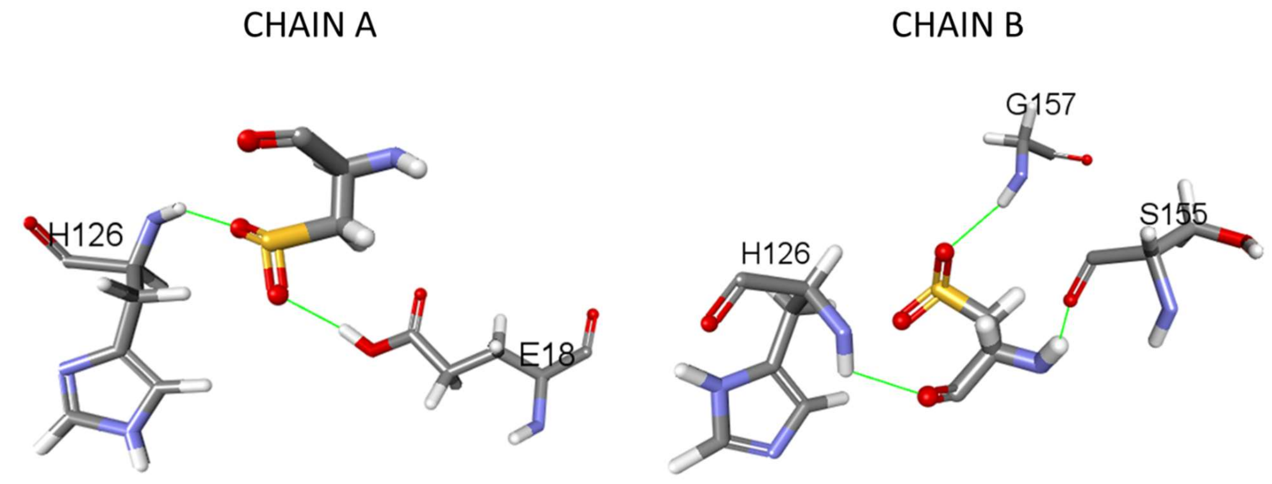

Figure S13. Differences in the representative interfacial contacts between the persulfidated and sulfinylated forms of DJ-1 – Average distances (Å) recorded over the last 45 ns of MD simulations as described above for the persulfidated (red) and sulfinylated (green) forms of DJ-1.

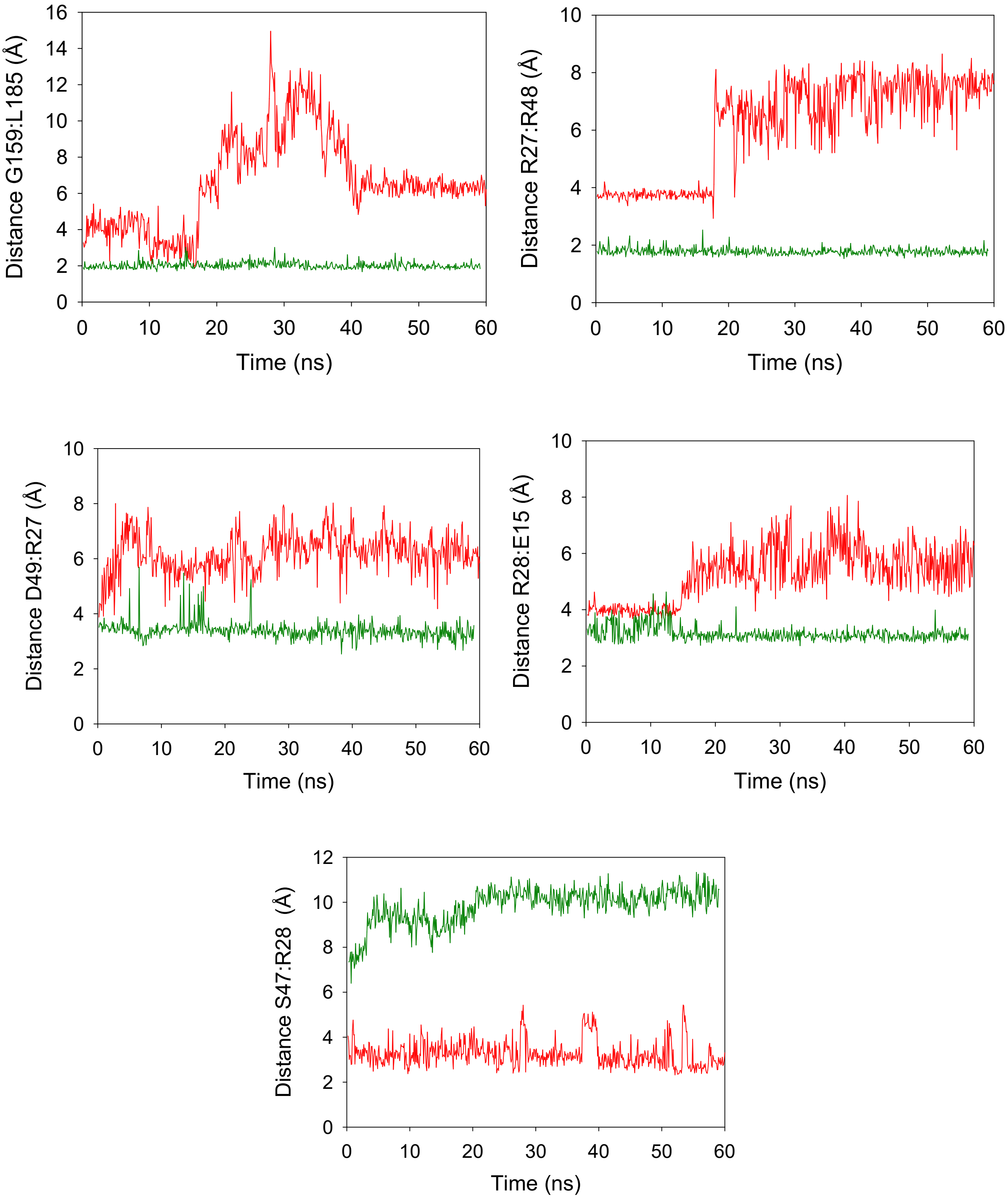

Figure S14. Persulfidation and sulfinylation induces different changes at the protein surface – Structural comparison of the surfaces from the wt, sulfinylated and persulfidated forms of DJ-1. Electrostatic surface representation (left) and hydrophobicity surface (right) for subunits A, B of wt and SS-DJ1 after MD simulations. Electrostatic potential is colored to show negative charge in red and positive charge in blue. Hydrophobic and hydrophilic regions are representated in brown and blue, respectively. The localization of the protein subunits and of the sulfur atoms (yellow balls) are shown in the middle.

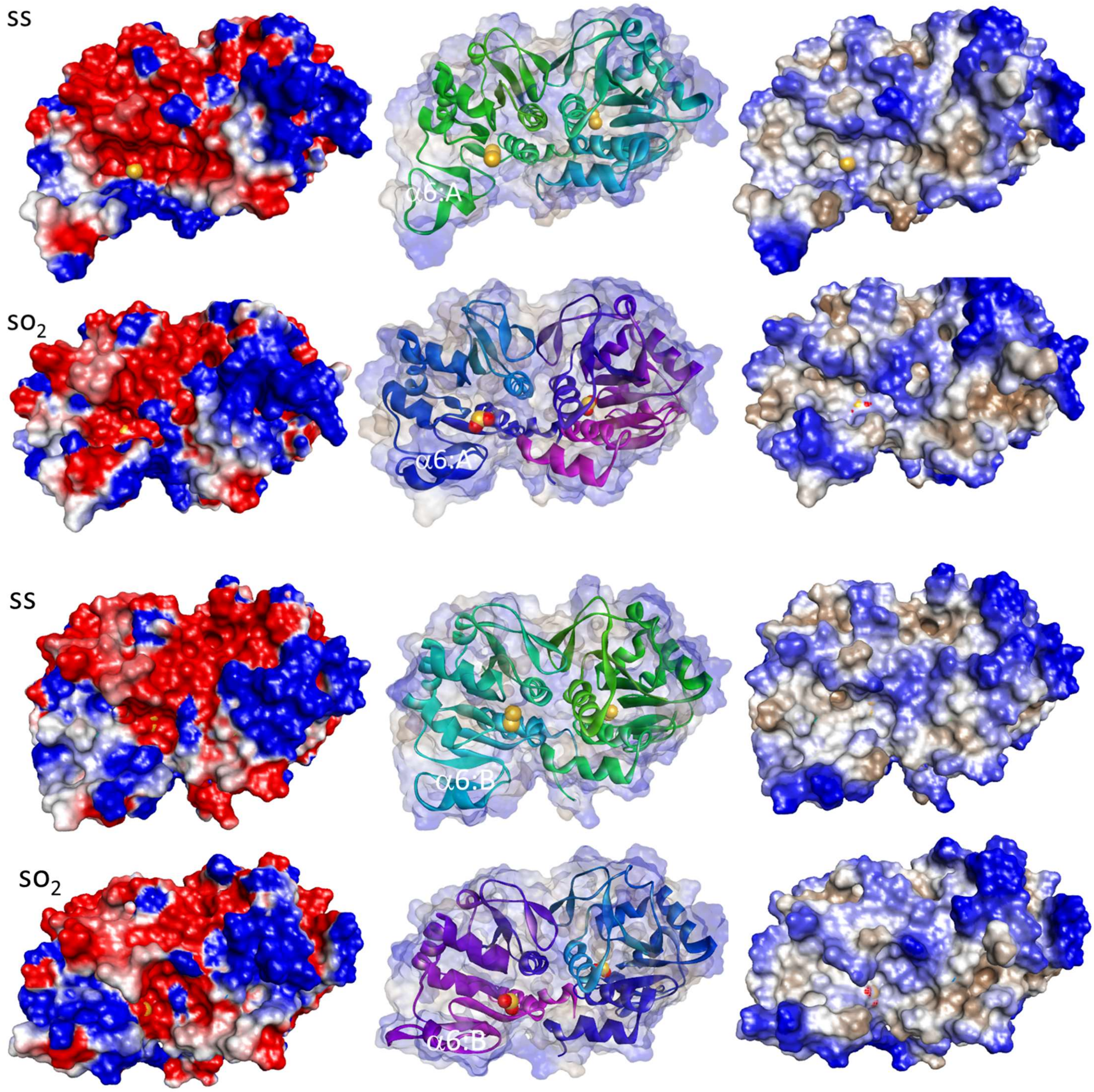

Supplement: Supplementary file 1 [file biomolecules-13-00027-s001.zip › biomolecules-2055187-supplementary.pdf]
